# Investigation into the correlation between humanistic care ability and emotional intelligence of hospital staff

**DOI:** 10.1186/s12913-022-08227-4

**Published:** 2022-06-30

**Authors:** Jingjing Ma, Wentao Peng, Jihong Pan

**Affiliations:** 1 Department of Pediatric Hematology Oncology Nursing, West China Second University Hospital, Sichuan University/West China School of Nursing, Sichuan University, Chengdu, Sichuan China; 2 Key Laboratory of Birth Defects and Related Diseases of Women and Children (Sichuan University), Ministry of Education, Chengdu, Sichuan China; 3 Department of Nursing, West China Second University Hospital, Sichuan University/West China School of Nursing, Sichuan University, Chengdu, Sichuan, China

**Keywords:** Hospital staff, Emotional intelligence, Humanistic care ability

## Abstract

**Background:**

There are different degrees of flaws in the knowledge structure of humanistic medicine of medical staff. The level of emotional intelligence of medical staff affects their career development as well as their relationship with patients. Currently, the research on humanistic care ability (HCA) and emotional intelligence of medical staff in China and other countries is rare. This study aimed to investigate the correlation between the level of HCA and level of emotional intelligence of the whole hospital staff.

**Methods:**

The questionnaire survey employed contained self-designed questions on the hospital staff members’ socio-demographic background, Caring Ability Inventory, and Wong and Law Emotional Intelligence Scale. The survey was conducted with the staff of West China Second University Hospital, Sichuan University in April 2020.

**Results:**

The hospital staff’s average CAI score was 197.77 ± 20.30, and their average WLEIS score was 84.21 ± 13.48. The CAI and WLEIS scores of the hospital staff who chose their college majors on their own interests were higher than those who chose their majors for other reasons (employability, suggestions from family or others, etc.). The CAI and WLEIS scores of the hospital staff who had received more comprehensive and in-depth humanistic care training were higher than those who did not. The CAI score of the hospital staff who had participated in volunteer service activities was higher than those who did not. The WLEIS score of the Pediatrics Department staff was higher than that of the Outpatient and Emergency Department staff, and the difference was statistically significant (*P* < 0.05). The scores of emotional intelligence, self-emotion assessment and expression, self-emotion management, self-emotion utilization, emotion recognition of others, and HCA of the hospital staff were positively correlated (*P* < 0.001).

**Conclusion:**

There were different levels of development of internal factors of emotional intelligence among the hospital staff, and their humanistic care ability was at a low level. Emotional intelligence was positively correlated to humanistic care ability. The findings suggest in-service training and education by healthcare institutions to enhance healthcare staff’s emotional intelligence for promoting the general health of the population.

## Background

The term “Care” is an important concept in the healthcare profession [1]. This outwardly affectionate behavioral action is contingent on the internal properties of cognition, practice, and education, an integration of knowledge, attitude, emotion, and behavior, all of which translate into conscientious service [2]. Empathy is a necessary personality and psychological trait of medical staff. Because the primary goal of medical personnel is to care for patients, a humanistic and altruistic mindset is expected of them at all times. Humanistic behavior is a prerequisite for social development and harmonious nurse-patient relationships; however, particularly in China, the humanistic characteristics that are necessary for giving effective care are frequently disregarded at management level [3]. The results of Chen and Liu [4] show that there were different degrees of flaws in the knowledge structure of humanistic medicine among medical staff in China. Further, humanistic care ability (HCA) is influenced by many factors, including personal character, social support, psychological characteristics, and work-related factors [5], and so HCA training therefore extends beyond the acquisition of medical knowledge.

Emotional intelligence refers to the ability of an individual to monitor his/her as well as others’ emotions, and to use this information to guide his /her thoughts and behavior [6]. Among healthcare practitioners, emotional intelligence is a necessary trait and skill. Salovey and Mayer [7] defined emotional intelligence as a “subset of social intelligence, including monitoring of one’s own and others’ feelings and emotions”. The ability to detect, use, comprehend, and manage one’s emotions can facilitate the enhancement of problem-solving ability in one’s emotional life. In recent years, there has been a gradual increase in the investigation of emotional intelligence in management, leadership, organizational behavior, and other fields. The influence of emotional intelligence on leadership, work pressure, work performance, turnover rate, self-concept, and other variables including commitment, morale, and health has been confirmed [8]. Rochester et al. [9] state that health workers must understand their own and others’ emotions to successfully interact with patients, cope with stress and uncertainty, and improve treatment outcomes. Other studies [10] have also shown that the level of emotional intelligence of the medical staff affects their career development and their relationship with patients. Individuals with higher emotional intelligence can better deal with highly emotional work (the art of managing one’s feelings) and are more likely to form a good doctor-patient and nurse-patient relationship. Weng [11, 12] showed that doctors with higher emotional intelligence were more likely to gain trust from patients. A study in South Korea [13] employed the dimensions of emotional perception, emotional expression, and empathy in emotional intelligence for the prediction of the doctor-patient relationship. Compared with doctors having negative emotions, such as irritability, anxiety and depression, those with high emotional intelligence could better respond, understand, and feel patients’ inner emotional experience from patients’ perspective, and were more likely to resonate with their patients [14]. As well as improving doctor-patient communication and promoting the doctor-patient relationship, the cognitive and regulatory abilities inherent in emotional intelligence also play an important role in improving clinical diagnosis, treatment and promoting patients’ health [15]. For clinical nurses, the perception of patients’ emotions is inseparable from exploring patients’ needs. Nurses with highly emotional perceptions are more likely to avoid nurse-patient conflict. In addition, emotional intelligence has also been found to be critical for managing the relationship between stress and mental health. People with poor emotional control tend to refuse the help of family, friends and others due to emotional problems, and so bear greater mental pressure [16]. Healthcare professionals often face pressure in their clinical work of consolidating and maintaining the health of patients. When coping with stress and fear, those with high self-perception and emotional intelligence display strong levels of engagement and attain great performance. This connection has recently been demonstrated by the results of a cross-sectional study conducted in several Spanish hospitals shortly after the COVID-19 outbreak in 2020 [17]. Therefore, it is necessary to proactively develop the emotional competencies of the healthcare workforce, especially in high-emotional demand contexts.

Currently, research on HCA and emotional intelligence worldwide is focused mainly on nurses and medical college students, including medical students and nursing students [18–22]. For example, Simmons and Cavanaugh [23] surveyed the HCA of female nursing graduates in the United States; Hables [24] conducted a cross-sectional CAI survey on nurses in the United States; Xu et al. [25] conducted a predictors-based CAI survey on nurses in China; Cho et al. [26], Hiroshi and Kohsuke [27] and Li et al. [28] surveyed the emotional intelligence of undergraduates. In addition, there have been a number of studies on the CAI evaluation of family caregivers of patients with chronic diseases in countries outside China [29]; Wan and Noor [30] conducted a study on the emotional intelligence of administrators. In short, the canon of knowledge on the HCA and emotional intelligence of the whole hospital staff is relatively sparse.

This study on the correlation between the level of HCA and level of emotional intelligence focused on the whole hospital staff including nurses, doctors, and technicians. We hypothesized that the hospital staff who had a high level of emotional intelligence had a high level of HCA; there were different levels of emotional intelligence and HCA among the hospital staff with different job titles; the hospital staff who received comprehensive and in-depth humanistic care training had a high level of HCA compared with those who lacked such training; the hospital staff were able to recognize their own emotions; and the hospital staff of different genders had different levels of HCA.

## Methods

### Ethics approval

This study was conducted in accordance with the Declaration of Helsinki as well as relevant guidelines and regulations. Ethical approval of this study was obtained from the Medical Ethics Committee of West China Second University Hospital, Sichuan University (Medical Research 2021 Ethics Approval No.077).

### Participants

The staff of West China Second University Hospital, Sichuan University was invited to participate in this cross-sectional study in April 2020. The hospital, having 1580 beds and 3641 staff members, is the largest women’s and children’s hospital in Southwest China. Because many factors may affect the hospital staff’s emotional intelligence and HCA, the results of this study are representative of similar hospitals to some extent.

### Survey tools

The questionnaire survey employed contained self-designed questions on the hospital staff members’ socio-demographic background, Caring Ability Inventory (CAI), and Wong and Law Emotional Intelligence Scale (WLEIS).

The socio-demographic part was used to collect information about each respondent’s gender, age, nationality, religious belief, education background, marital status, working years, occupation, professional title, department, family address, whether the respondent was his/her parents’ only child, reasons for choosing the relevant university/college major, whether he/she had received training related to humanistic care, and whether he/she had participated in voluntary service activities.

Caring ability Inventory (CAI) is a self-reported measurement method used to test a person’s ability to care for others. The scale was developed by Ngozi Nkongho [31]; the tool has passed various reliability and validity tests, and has since been used in several academic and clinical environments in China and worldwide [32, 33]. Yulian Ma, a Chinese scholar, translated the scale [34] into Chinese and demonstrated the sound psychometric properties of the scale, which consisted of 37 items grouped into 3 dimensions: knowing (how well you know yourself, others, and your surroundings; 14 items), courage (ability to handle unknown situations; 13 items), and patience (endurance and toughness; 10 items). The Likert 7-level scoring method was used to score each response, from “totally disagree” to “totally agree” with 1–7 points, respectively; some items were reverse scored. The Cronbach’s alpha was 0.919, the content validity index was 0.957, and the score ranged from 37 to 259. The HCA can be measured using the CAI score. The higher the CAI score, the higher level of HCA.

The Wong and Law Emotional Intelligence Scale (WLEIS) was developed in Hong Kong in 2002 by Wong [35]. Since then, it has been used in many countries. Cross-cultural studies employing the WLEIS have been carried out in many countries, such as Portugal [36], Japan [27], and South Korea [37]. The Chinese version of WLEIS is also widely used in mainland China. WLEIS has 4 dimensions, and each dimension has 4 items; thus, the scale comprises a total of 16 items, including self-emotion assessment and expression (which refers to the ability of everyone to understand their deep emotions and express them naturally), self-emotion management (which refers to the ability of people to regulate their emotions, so that they can recover from emotional fluctuations and anxiety more quickly), self-emotion utilization (which refers to the ability of individuals to use their emotions to guide themselves to constructive activities and personal performance), and emotion recognition of others (which refers to the ability of individuals to perceive and understand the emotions of people around them) [38]. The Likert 7-level scoring method was used, whereby 1–7 points were given (from “completely disagree” to “completely agree”). Regarding the internal consistency of each dimension, Cronbach’s alpha values ranged between 0.76 and 0.89, and the score ranges between 16 and 112. Emotional intelligence can therefore be measured reliably using the WLEIS score. The higher the WLEIS score, the higher the level of emotional intelligence.

### Data collection

The researchers created a standardized questionnaire with instructions and guidelines that was completed anonymously by respondents using the hospital’s WeChat platform. The respondents were not required to provide their personally identifiable information (name, identify card, or contact information). During the survey, the participants were informed of the aim and significance of this study and were required to complete the questionnaire voluntarily. The completion of the questionnaire was regarded as the respondent’s verbal consent to participate in the study. The questionnaire was produced through Wenjuanxing, an online questionnaire survey tool. The researchers could only access the questionnaire data by logging into their Wenjuanxing user account with a password, so the researchers ensured the survey anonymity and confidentiality. A total of 3641 questionnaires were distributed, and 769 were returned, thus the recovery rate was 21.12%.

### Statistical methods

The SPSS 22.0 statistical program was used for data analysis. The descriptive statistics for CAI and WLEIS scores had a normal distribution and were calculated as mean ± standard deviation (SD). Independent t-test and one-way analysis of variance (ANOVA) were used to investigate variations in caring abilities among individuals based on socio-demographic factors. The Least Significant Difference (LSD) method for comparison of the two scales was utilized to calculate the degree of correlation between scales, based on the Pearson correlation method. Independent variables (*P* < 0.05) in the univariate analysis were entered into the multivariate analyses. The predictors of caring abilities were identified using stepwise multiple linear regression. The two-sided level α = 0.05 was used, and the significance threshold was established at *P* < 0.05.

## Results

### Socio-demographic characteristics of hospital staff

The general information collected from hospital staff members who participated in this study is presented in Table 1.

**Table 1:** Socio-demographic characteristics of hospital staff (*n* = 769)

Variables	N	%
**Gender**		
Male	85	11.05
Female	684	88.95
**Ethnicity**		
Han ethnic group	750	97.53
Other	19	2.47
**Are you the only child of your parents?**		
No	430	55.92
Yes	339	44.08
**Religious beliefs**		
None	760	98.83
Yes	9	1.17
**Education background**		
Junior college	107	13.91
Undergraduate	457	59.43
Postgraduate	205	26.66
**Occupation**		
Nurse	332	43.17
Doctor	148	19.25
Technician	187	24.32
Other	102	13.26
**Marital status**		
Married	449	58.39
Unmarried or divorced or widowed	320	41.61
**Professional title**		
Junior title	542	70.48
Intermediate title	183	23.80
Deputy senior or senior professional title	44	5.72
**Department**		
Pediatrics	232	30.17
Obstetrics and Gynecology	191	24.84
Outpatient and Emergency	111	14.43
Medical Technology	179	23.28
Other	56	7.28
**Family residence**		
City or town	536	69.70
Rural areas	233	30.30
**Reasons for choosing the relevant university/college major**		
Self-interest	300	39.01
Employability	83	10.79
Suggestions from family or others	358	46.55
Other	28	3.64
**Have you received training on humanistic care?**		
No relevant training	106	13.78
Less training	515	66.97
More comprehensive and in-depth	148	19.25
**Have you ever participated in volunteer service?**		
No	208	27.05
Yes	561	72.95

### CAI and WLEIS scores of hospital staff

The emotional intelligence score (average WLEIS score) was 84.21 ± 13.48. The average CAI score was 197.77 ± 20.3, of which 332 nurses scored 198.71 ± 18.73. Compared with the international norms of nurse care ability [39], it was at a lower level (Tables 2 and 3).

**Table 2:** CAI and WLEIS scores of hospital staff (*n* = 769) ($\overline{x }\pm s$)

Items	Average dimensions or items
**Humanistic care ability**	
**Knowing**	78.25 ± 9.77 (5.59 ± 0.70)
CAI 2- today’s society is full of opportunities	6.17 ± 1.17
CAI 3- what I say is usually what I think	5.65 ± 1.18
CAI 6- even if people don’t like me, I can still like them	4.59 ± 1.47
CAI 7- I’m easy to understand	5.68 ± 1.02
CAI 9- I’m willing to take the time to get to know people	5.39 ± 1.21
CAI 19- people think I’m a man of my word	6.22 ± 0.89
CAI 22- I find that everything has its meaning	6.18 ± 1.07
CAI 26 I really like myself	5.48 ± 1.17
CAI 30- I can accept all kinds of people	4.54 ± 1.48
CAI 31- when caring for others, I never hide my feelings	5.03 ± 1.34
CAI 33- I can express my feelings to others in a warm and caring way	5.46 ± 1.19
CAI 34- I love talking to people	5.37 ± 1.3
CAI 35- I think I’m sincere in dealing with others	6.14 ± 0.89
CAI 36- people need private space to think and feel	6.36 ± 0.87
**Courage**	58.71 ± 11.07 (4.52 ± 0.85)
CAI 4- there’s nothing I can do for a desperate person	4.26 ± 1.63
CAI 8- in terms of what I need to know, I already know enough	5.05 ± 1.51
CAI 11- I can’t make life better	5.55 ± 1.44
CAI 12- I often feel uneasy when others depend on me	5.23 ± 1.48
CAI 13- I don’t want to interrupt my business to help others	4.93 ± 1.47
CAI 14- it’s hard for me to express my feelings when I get along with others	5.01 ± 1.53
CAI 15- I only care about doing things right, no matter what they say	5.03 ± 1.65
CAI 16- I find it hard to understand people with similar experiences	4.84 ± 1.59
CAI 23- I find it hard to understand people without similar experiences	3.22 ± 1.66
CAI 25- I’m not willing to make promises I can’t fulfill	2.02 ± 1.18
CAI 28- new experiences often frighten me	4.19 ± 1.59
CAI 29- I’m afraid to let others know me publicly	4.23 ± 1.59
CAI 32- I don’t like people asking me for help	5.15 ± 1.33
**Patience**	60.81 ± 5.35 (6.08 ± 0.54)
CAI 1- I think learning is cumulative	6.75 ± 0.75
CAI 5- I think I still need to improve	6.63 ± 0.66
CAI 10- sometimes I want to care about others, sometimes I don’t want to care about others	4.96 ± 1.44
CAI 17- I admire those who are calm and patient	6.31 ± 0.91
CAI 18- I think it’s important to respect and accept the opinions and feelings of others	6.44 ± 0.8
CAI 20- I think it’s not important to respect and accept the opinions and feelings of others	6.54 ± 0.74
CAI 21- good friends should take care of each other	6.54 ± 0.74
CAI 24- I like to encourage people	5.93 ± 1.01
CAI 27- I can see everyone’s strengths and weaknesses	5.24 ± 1.19
CAI 37- it’s easy for people to get along with me at any time	5.48 ± 1.17
**Total score**	**197.77 ± 20.3 (5.35 ± 0.55)**
**Emotional intelligence**	
**Self-emotion assessment and expression**	23.06 ± 3.63 (5.76 ± 0.91)
WLEIS 1- usually I know why I feel something	5.74 ± 1.01
WLEIS 2- I know my emotions very well	5.75 ± 1.03
WLEIS 3- I really understand how I feel	5.77 ± 1.01
WLEIS 4- I often know why I feel happy or unhappy	5.8 ± 1.06
**Self-emotion management**	19.91 ± 4.61 (4.98 ± 1.15)
WLEIS 5- when I encounter difficulties, I can control my temper and solve problems rationally	5.12 ± 1.22
WLEIS 6- I can control my emotions	4.97 ± 1.29
WLEIS 7- when I’m angry, I usually calm down in a short time	4.95 ± 1.31
WLEIS 8- I have great control over my emotions	4.86 ± 1.34
**Self-emotion utilization**	20.84 ± 4.08 (5.21 ± 1.02)
WLEIS 9- I usually set goals for myself and try to achieve them	5.11 ± 1.25
WLEIS 10- I often tell myself that I am a very capable person	4.9 ± 1.24
WLEIS 11- I am a person who can encourage myself	5.42 ± 1.15
WLEIS 12- I always encourage myself to be the best	5.41 ± 1.15
**Emotion recognition of others**	20.41 ± 4.26 (5.21 ± 1.02)
WLEIS 13- I can usually guess a friend’s or colleague’s mood from their behavior	5.17 ± 1.13
WLEIS 14- I have a strong ability to observe other people’s emotions	5.11 ± 1.2
WLEIS 15- I have keen insight into other people’s feelings and emotions	5.12 ± 1.18
WLEIS 16- I know the emotions of people around me	5.01 ± 1.15
**Total score**	**84.21 ± 13.48 (5.26 ± 0.84)**

**Table 3:** Evaluation grade of the caring ability of international norm nurses

Scale dimensions	High	Medium	Low
Total score	> 220.30	220.30 ~ 203.10	< 203.10
knowing	> 83.040	83.04 ~ 63.40	< 63.40
Courage	> 69.740	69.74 ~ 48.88	< 48.88
Patience	> 64.680	64.68 ~ 52.34	< 52.34

### Comparison between different socio-demographic characteristics, and CAI and WLEIS scores

The results show that females on average had higher CAI score than males. The CAI and WLEIS scores of the hospital staff who chose college majors based on their own interests were higher, compared to the score of those who selected their majors for other reasons (employability, suggestions from family or others, etc.). Respondents who had received more comprehensive and in-depth humanistic care training or participated in volunteer service activities had higher CAI and WLEIS scores, compared with those who did not possess this training and experience. The average WLEIS score of the Pediatrics Department staff was higher than that of the Outpatient and Emergency Department staff, indicating a statistically significant difference (*P* < 0.05). There was no significant difference between CAI and WLEIS scores regarding other demographic characteristics (Table 4).

**Table 4:** Comparison of CAI and WLEIS scores of hospital staff with different socio-demographic characteristics (score, $\overline{x }\pm s$)

Variables	CAI			WLEIS		
	Score	t/F	*P*	score	t/F	*P*
**Gender**						
Male	192.26 ± 20.81	-2.66	0.008	83.67 ± 14.48	-0.39	0.694
Female	198.45 ± 20.14			84.28 ± 13.36		
**Department**						
Pediatrics	199.73 ± 19.52	1.1	0.354	86.38 ± 12.95^a^	2.71	0.029
Obstetrics and Gynecology	197.32 ± 18.89			83.88 ± 13.28		
Outpatient and Emergency	195.02 ± 21.22			81.77 ± 13.94^a^		
Medical Technology	197.31 ± 22.31			83.78 ± 13.87		
Other	198.09 ± 19.39			82.63 ± 13.30		
**Reasons for choosing this major**						
Self-interest	200.66 ± 21.23^ab^	3.41	0.017	86.41 ± 13.68^abc^	4.72	0.003
Employability	195.25 ± 18.84^a^			82.8 ± 14.96^a^		
Suggestions from family or others	196 ± 19.32^b^			82.99 ± 12.65^b^		
Other	196.82 ± 23.58			80.54 ± 14.37^c^		
**Have you received training on humanistic care**						
No relevant training	194.97 ± 18.96^a^	15.94	< 0.0001	83.16 ± 13.29^a^	35.27	< 0.0001
Less training	195.97 ± 19.54^b^			82.14 ± 12.50^b^		
More comprehensive and in-depth	206.05 ± 21.79^ab^			92.2 ± 14.03^ab^		
**Have you ever participated in volunteer service**						
No	194.38 ± 21.09	-2.83	0.005	82.73 ± 13.27	-1.86	0.063
yes	199.03 ± 19.86			84.76 ± 13.53		

*Note*: LSD method was used for pairwise comparison. In the same dimension, the two groups marked with a at the same time indicate that the difference between the two groups was statistically significant (*P* < 0.05), the two groups marked with b at the same time indicate that the difference between the two groups was statistically significant (*P* < 0.05), the two groups marked with c at the same time indicate that the difference between the two groups was statistically significant (*P* < 0.05). The t value was for two groups of variables and the F value was for multiple groups of variables

### Correlation analysis of hospital staff’s CAI and WLEIS scores

The Pearson correlation analysis showed a significantly positive correlation between the scores of emotional intelligence, self-emotion evaluation and expression, self-emotion management, self-emotion application, others’ emotion recognition, and HCA of hospital staff (*P* < 0.001) (Table 5).

**Table 5:** Correlation Analysis of hospital staff’s CAI and WLEIS scores (*n* = 769)

Items	Knowing	Courage	Patience	CAI score
Self-emotion assessment and expression				
*R*	0.622	0.253	0.561	0.585
*P*	< 0.0001	< 0.0001	< 0.0001	< 0.0001
Self-emotion management				
*R*	0.567	0.234	0.343	0.491
*P*	< 0.0001	< 0.0001	< 0.0001	< 0.0001
Self-emotion utilization				
*R*	0.687	0.256	0.459	0.592
*P*	< 0.0001	< 0.0001	< 0.0001	< 0.0001
Emotion recognition of others				
*R*	0.535	0.185	0.396	0.463
*P*	< 0.0001	< 0.0001	< 0.0001	< 0.0001
WLEIS score				
*R*	0.739	0.284	0.533	0.651
*P*	< 0.0001	< 0.0001	< 0.0001	< 0.0001

Table 6 represents the result of multiple regression analysis in predicting three dimensions and overall caring ability. The results indicated the existence of positive correlations among the Intercept (± SD6.896, t = 16.200, *P* = 0.0001, CI = 95%), self-emotion assessment and expression (± SD9.460, t = 1.391, *P* = 0.0001, CI = 95%,), self-emotion management (± SD1.116, t = 5.640, *P* = 0.0001, CI = 95%,), and emotion recognition of others (± SD0.597, t = 0.281, *P* = 0.000, CI = 95%,).

**Table 6:** Caring Ability Inventory multiple linear regression analysis

Variable	Estimate	Standard error	t value	*P*-value	95% confidence interval	
					Lower limits	Upper limits
Intercept	111.750	6.896	16.200	< 0.0001	98.211	125.289
Age	0.125	0.097	1.290	0.199	-0.066	0.315
Gender (male)	-2.946	1.708	-1.72	0.085	-6.3	0.408
Education (undergraduate)	-0.083	1.603	-0.050	0.959	-3.230	3.064
Education (postgraduate)	-1.092	2.337	-0.470	0.641	-5.680	3.497
Occupation (nurse)	1.157	1.837	0.630	0.529	-2.450	4.763
Occupation (doctor)	5.178	2.487	2.080	0.038	0.296	10.060
Occupation (technician)	2.489	2.231	1.120	0.265	-1.890	6.869
Working years (≥ 5 years, < 10 years)	-1.310	1.451	-0.900	0.367	-4.158	1.539
Working years (≥ 10 years)	-0.921	2.015	-0.460	0.648	-4.877	3.035
Marital status (unmarried or divorced or widowed)	0.414	1.339	0.310	0.757	-2.215	3.043
Are you the only child of your parents (yes)	-0.014	1.090	-0.010	0.990	-2.155	2.126
Religious beliefs (yes)	-4.232	4.821	-0.880	0.380	-13.696	5.233
Family residence (city or town)	-0.109	1.267	-0.090	0.932	-2.595	2.378
Title (intermediate title)	-2.101	1.733	-1.210	0.226	-5.503	1.300
Title (deputy senior or senior professional title)	-7.243	3.038	-2.380	0.017	-13.206	-1.279
Department (pediatrics)	-1.841	2.160	-0.850	0.394	-6.081	2.399
Department (obstetrics and gynecology)	-1.681	2.177	-0.770	0.440	-5.954	2.592
Department (outpatient and emergency)	-2.110	2.391	-0.880	0.378	-6.804	2.585
Department (medical technology)	-1.145	2.425	-0.470	0.637	-5.906	3.616
Reasons for choosing the major (self-interest)	0.050	2.916	0.020	0.986	-5.674	5.774
Reasons for choosing this major (employability)	-0.186	3.199	-0.060	0.954	-6.466	6.094
Reasons for choosing this major (suggestions from family or others)	0.209	2.894	0.070	0.942	-5.472	5.890
Have you received training on humanistic care (less training)	1.071	1.576	0.680	0.497	-2.023	4.164
Have you received training on humanistic care (more comprehensive and in-depth)	1.933	1.909	1.010	0.312	-1.815	5.682
Have you ever participated in volunteer service (yes)	2.373	1.227	1.930	0.054	-0.035	4.781
Self-emotion assessment and expression	1.755	0.186	9.460	< 0.0001	1.391	2.119
Self-emotion management	0.162	0.161	1.000	0.316	-0.155	0.478
Self-emotion utilization	1.116	0.198	5.640	< 0.0001	0.728	1.504
Emotion recognition of others	0.597	0.161	3.710	0.000	0.281	0.913

The results show that age, professional title, participation in the humanistic care activities for patients, self-emotion assessment and expression, self-emotion utilization, and emotion recognition of others had statistically significant effects on CAI score.

## Discussion

This study reveals variations among the hospital staff in terms of development of internal factors of emotional intelligence. Among them, HCA was at a relatively low level, and emotional intelligence was found to be positively correlated to HCA. The average CAI score was 197.77 ± 20.3, lower than the international norm (198.79 being the lower level among nurses), but higher than that reported by Xu et al. [25], Chi et al. [20], Ge et al. [18], and Zhao et al. [19]. The CAI score lower than the international norm might be ascribed to different cultural backgrounds and educational systems. In traditional Chinese culture, care tends to occur only in intimate relationships, while medical care for patients is implicit. The low average CAI score might also be a reflection of the low premium attached to service and humanistic care in Chinese medical education. On the other hand, Western medical education is known to pay more attention to practical care education for medical students, resulting in higher CAI scores.

In general, in Western countries there is more academic research on the HCA of medical students. For example, the survey results of Labrague et al. [40] for 167 medical students from four countries show they generally exhibited positive caring behavior. The research results of Cheng et al. [41] show that the total CAI score and each dimension score of Chinese medical students are significantly lower than those of American medical students. Fjortof et al. [42]. investigated the caring ability of 323 pharmacists in the United States. Their CAI score was 203.0 ± 17.7, which was not a high level. There exist sizeable differences in terms of CAI scores between medical and healthcare personnel in different countries. It should also be noted that pharmacists’ CAI scores are not directly comparable with those of medical and healthcare personnel.

In this study, the average score of each dimension of CAI (from high to low) (knowing, patience, and courage) indicate that the hospital staff had a relatively good understanding of themselves, others, and the surrounding environment. Also, their patience (endurance and toughness) score was similar to the research results of Wessel [43] and the research results of Xu [25], and their score for courage was significantly higher than that reported by Xu [25]. The scores for understandings and patience, and the total score of this study sample were higher than those reported by He et al. [44]. These differences exist probably because Xu [25] and He et al. [44] only studied nursing staff, while this study was conducted on the whole hospital staff. Caring is multidimensional, and caring behavior can be taught and learned [32]. Unlike China, in many foreign countries the provision of medical humanistic education has existed for many years. Bevis and Watson [45] established humanistic care courses back in 1989. Tomura et al. [46] pointed out that humanistic care education should focus on the distinctly human elements of appreciating nature, as well as science, art and relationship. Currently, medical humanities courses account for 20–30% of total class hours in the United States, and about 10–15% in the United Kingdom [47]. In terms of teaching and evaluation methods, foreign medical colleges have adopted various teaching methods and means, such as course lectures, group discussions, academic discussions, extracurricular reading, reflection diaries, and essay-writing. Arveklev [48] used improvisation, role play, and forum drama to improve students’ understanding of caring and to enable them to explore the caring experience. By comparison, there are some problems within the setting of medical humanities courses in China, such as insufficient resourcing, lack of unified standards, lack of coherence, and absence of cross-disciplinary course design. Regarding course content, humanities courses are mainly focused on ethics and psychology [49]. Further, in China the proportion of humanities courses is about 7–8%, which is relatively low compared with that in many foreign medical colleges. The teaching and evaluation methods are structured upon a large class teaching system. Teaching tends to be heavily theoretical, and thus lacking in flexibility and diversity. As observed in previous studies, there exist significant shortcomings in the humanistic care curriculum in China’s medical colleges, although in recent years, many practitioners in these institutions have taken steps to improve the curriculum [50]. Further, the proportion of humanistic care courses across medical colleges in China has increased, and the teaching styles are more diversified. However, there is still a long way to go before the educational effect of these innovations will impact on the HCA of clinical front-line medical and healthcare personnel.

This study has shown that the average score for the emotional intelligence of hospital staff was 84.21 ± 13.48, higher than the research results of Yu [51] and Wang et al. [52]. The scores of each dimension, ranked from high to low, are as follows: self-emotion assessment and expression (23.06 ± 3.63), self-emotion utilization (20.84 ± 4.08), emotion recognition of others (20.41 ± 4.26), and self-emotion management (19.91 ± 4.61). These results indicate different levels of development of internal factors of emotional intelligence among the hospital staff. Davies et al. [53] reported that regulation of emotion in oneself involved change of his/her mood. This may explains why the scores of self-emotion management in our study were low. A person’s mood may change all the time, so it is difficult to maintain emotional stability. This suggests that the hospital staff’s ability for self-emotion management needs to be improved through training programs, especially rapid correction of one’s emotions.

Our study has also shown that the hospital staff’s scores of self-emotion assessment and expression were higher than their scores of emotion recognition of others. This is similar to the research result of Acosta-Prado [38], which reported that Colombian managers had a stronger ability to evaluate their own emotions and a weaker ability to recognize the emotions of others. The population of our study is different from that of the study of Acosta-Prado [38], but the results are similar, indicating a possible common weakness in emotion recognition of others in different population groups. Emotional intelligence is not innate [54], and it can be improved by self-learning, environmental intervention, and other measures. Hospital management shall train staff on emotional intelligence, and stimulate the internal emotions of individuals, in order to help them better understand and predict the emotions and behaviors of others. The training mode can include education courses, such as listening training, self-management, interpersonal communication, and regular organization of lectures on emotional intelligence.

The results of this study also show that females possess higher HCA than males, a pattern which is consistent with that reported in several studies [18, 20]. These results may be indicative of women’s potentially higher patience, carefulness, empathy, and emotional sensitivity. The CAI and WLEIS scores of those who chose their majors based on their own interests were higher than those who had selected majors for other reasons (employability, suggestions from family or others, etc.). Regarding internal driving force, employees have different feelings and draw satisfaction from work for different reasons. In general, employees who feel satisfied with their jobs have a stronger ability to overcome difficulties [55]. Those who choose their majors and occupation voluntarily gain a greater sense of professional value and are more willing to express concern for patients [56]. Therefore, stimulating employees’ interest and love for their work in a variety of ways could improve their HCA and emotional intelligence. For example, the situational simulation training in doctor-patient communication could enable the doctor to communicate with the patient with transposition thinking, thereby making the doctor better understand the patient’s emotions during diagnosis and treatment. Hospital managers are advised to give the staff complete freedom to provide suggestions, thereby making the staff feel valued. The CAI and WLEIS scores were higher for staff who received more comprehensive and in-depth humanistic care training, compared with those who lacked such training. The study of Luo et al. [57] showed that emotional intelligence training for clinical nurses helped promote general health and improve the emotional intelligence of the trainees. Their study results were consistent with those in earlier studies [54, 58]. Moreover, researchers have found that medical students who participated in social practice more often have better humanistic care ability compared with non-participants [49]. Paige et al. [59] found that people can gain satisfaction from having a variety of social practice interaction opportunities. Practice is the source of cognition. In practice, care can be effectively practiced in the process of contacting patients, which can promote the improvement of humanistic care ability to a certain extent [60].

Volunteer activity is one form of clinical practice. The results of this study show that CAI scores of those who had participated in volunteer service activities were higher than those who had not. This is because volunteer servicing entails dedication, and, as a result, promotes humanistic care for others. In addition, these volunteers might have received relevant training in their activities and experienced the atmosphere of humanistic care, which may explain why their humanistic care scores were higher than those who did not participate [18, 20, 57].

In our study, the Pediatrics Department staff had higher WLEIS score than the Outpatient and Emergency Department staff, indicating different levels of emotional intelligence and HCA among the hospital staff. This may be due to long-term contact with pediatric patients, which can fortify feelings of empathy for children. Furthermore, pediatric staff work is not only for children, but also for their families. Due to the particularity of their work objects and the impact of work pressure, pediatric nurses pay more attention to the regulation of self-emotion, hence why the WLEIS scores of pediatric staff are higher [61].

In the correlation analysis, it was found that the hospital staff’s emotional intelligence was positively correlated with HCA. The higher the emotional intelligence score, the higher the HCA, a pattern which is consistent with the results of conclusions of Chi et al. [20]. Individuals with high emotional intelligence are more likely to observe emotional changes occurring in patients, family members, and colleagues around them, and are more capably of forming good interpersonal relationship with them. Individuals with low emotional intelligence are more self-centered, less aware of the feelings of people around them, and exhibit poorer humanistic care [51]. Emotional intelligence could stimulate the internal emotions of individuals into practical action, translating their emotions into humanistic care for patients.

### Implications for practice and future research

Improving the health status of individuals and population is a central ambition of healthcare systems in high-income countries. Emotional intelligence aids in analyzing doctor-patient communication, doctor-patient engagement, clinical diagnosis, and minimizing hospital staff-patient conflict, all of which benefit the healthcare system. Therefore, the findings of this study may have beneficial implications for medical practice and research. The overall level of HCA of hospital staff was relatively low. However, the CAI and WLEIS scores of the staff who received more comprehensive and in-depth humanistic care training were higher compared with the scores of other staff. These findings alone highlight the importance of developing and implementing training programs to improve the HCA and emotional intelligence of all hospital staff. Meanwhile, hospital management needs to strengthen the theoretical and practical education of humanistic care and improve the care awareness of hospital staff. Moreover, voluntary service activities and team psychological counseling can uplift the overall HCA of medical staff. Thus it is advised to better adapt and train the hospital staff on voluntary service activities to uplift general health and improve the emotional intelligence of hospital staff.

### Limitations

This study has several limitations. First, we only selected one hospital for investigation. Although this hospital allows for large-scale, high-grade, convenient sampling, and is reasonably representative of such hospitals across Southwest China, the universality of our research results at both national and international level is limited. Second, the cross-sectional design of the study limited the inference of causality between variables; thus, it is better to conduct longitudinal research and further explore more targeted predictors of HCA. Third, the health care outcomes of HCA from patients’ perspectives in the study are not covered. There is insufficient research on the differences between HCA and emotional intelligence of all kinds of medical personnel. It would be helpful for future studies to comprise in-depth investigation of more appropriate methods for improving humanistic care ability and emotional intelligence.

## Conclusion

In summary, the hospital staff’s levels of emotional intelligence affects their levels of HCA. It may be argued that the internal factors of emotional intelligence among the hospital staff are at different levels of development, with many of them exhibiting low HCA. Therefore, it is necessary to provide more emotional intelligence training for hospital staff to improve their HCA. Implementation of humanistic teaching activities closely combined with clinical practice through the design and training of the knowledge structure construction module of humanistic medicine is needed to improve the hospital staff’s humanistic knowledge structure, communication ability, and HCA.

## Data Availability

The datasets generated and/or analyzed during the current study are not publicly available due to privacy guidelines but are available from the corresponding author on reasonable request.

## Abbreviations

CAI: Caring Ability Inventory
HCA: Humanistic Care Ability
LSD: Least Significant Difference
SD: Standard Deviation
WLEIS: Wong and Law Emotional Intelligence Scale

## References

[1] Warelow P, Edward KL (2007). Caring as a resilient practice in mental health nursing. Int J Ment Health Nurs.

[2] Chen Y (2017). Caring ability among Chinese nursing students: a cross-sectional study and educational intervention.

[3] Shin S, Kang J (2019). Development and validation of a person-centered perioperative nursing scale. Asian Nurs Res.

[4] Chen YX, Liu H (2015). Investigation on the cognition of knowledge structure of humanistic medicine. Med Philos.

[5] Kovner C, Brewer C, Wu YW, Chen Y, Suzuki M (2006). Factors associated with work satisfaction of registered nurses. J Nurs Scholarsh.

[6] Lv HJ, Han CX, Wang DJ (2018). Meta analysis of the influence of leader’s emotional intelligence on leadership effectiveness. Adv Psychol Sci Exhibition.

[7] Salovey P, Mayer JD (1990). Emotional intelligence. Imagin Cogn Pers.

[8] Landa JMA, López-Zafra E (2010). The impact of emotional intelligence on nursing: an overview. Psychology.

[9] Rochester S, Kilstoff K, Scott G (2005). Learning from success: improving undergraduate education through understanding the capabilities of successful nurse graduates. Nurse Educ Today.

[10] Bai JM (2014). The Effect about positive and negative emotion, emotional intelligence to emotional labor strategies for medical students.

[11] Weng HC (2008). Does the physician’s emotional intelligence matter? Impacts of the physician’s emotional intelligence on the trust, patient-physician relationship, and satisfaction. Health Care Manage Rev.

[12] Weng HC, Steed JF, Yu SW, Liu YT, Hsu CC, Yu TJ (2011). The effect of surgeon empathy and emotional intelligence on patient satisfaction. Adv Health Sci Educ Theory Pract.

[13] Kim SH, Ko JK, Park JH (2011). Effect of emotional intelligence on patient-physician interaction scores of clinical performance examination. Korean J Med Educ.

[14] Wang XR, Wang MF, Li XX, Liang R (2020). Influence of emotional intelligence on clinical communication ability of nursing interns. Nurs Res.

[15] Pu FF, Bai YH, Wang P, Zhou L, Guo XL (2017). The application of emotional intelligence in the study of doctor-patient relationship. Med Philosophy.

[16] Yan YM, Gong M (2015). Advances of emotional intelligence in nursing organizations. J Shanghai Jiaotong Univ Med Sci.

[17] Sanchez-Gomez M, Sadovyy M, Breso E (2021). Health-care professionals amid the COVID-19 pandemic: how emotional intelligence may enhance work performance traversing the mediating role of work engagement. J Clin Med.

[18] Ge XH, Li JS, Chen HY (2014). Analysis of humanistic care ability of medical students and its influencing factors. Shanghai Jiaotong Univ Med Sci.

[19] Zhao L, Zeng Y, Liao L, Wang SM, Yang YH, Xia YP (2019). Path analysis of the influence of empathy and interpersonal communication on humanistic care ability among undergraduate nursing students. Nurs Res.

[20] Chi Y, Wang JJ, Li HY (2020). Intermediary effect of emotional between empathy and humanistic care of nursing students. Nurs J Chin PLA.

[21] Guo T, Sui SJ, Yu F, Yao HB (2015). Research progress of nurses’ emotional intelligence training. J Nurs Educ.

[22] Xie C, Li X, Zeng Y, Hu X (2021). Mindfulness, emotional intelligence and occupational burnout in intensive care nurses: a mediating effect model. J Nurs Manag.

[23] Simmons PR, Cavanaugh SH (2000). Relationships among student and graduate caring ability and professional school climate. J Prof Nurs.

[24] Hables RM (2013). Factors associated with caring abilities among nurses working at El-Shatby maternity university hospital. J Am Sci.

[25] Xu T, Wang Y, Wang R, Lamb KV, Ren D, Dai G (2021). Predictors of caring ability and its dimensions among nurses in China: A cross-sectional study. Scand J Caring Sci..

[26] Cho S, Drasgow F, Cao M (2015). An investigation of emotional intelligence measures using item response theory. Psychol Assess.

[27] Hiroshi T, Kohsuke Y (2011). Development of a Japanese version of Wong and law emotional intelligence scale. Bull Center Educ Res Dev.

[28] Li TW, Saklofske DH, Bowden SC, Yan GG, Fung TS (2012). The measurement invariance of the Wong and law emotional intelligence scale (WLEIS) across three chinese university student groups from Canada and China. J Psychoeduc Assess.

[29] Barrera L, Carrillo GM, Chaparro L, Pinto N, Rodríguez A, Sánchez B (2011). Effect of the program caring for caretakers»: findings of a multicenter study. Colombia Méd.

[30] Wan S, Noor M (2015). Examining the psychometric properties of the Wong and law emotional intelligences scale (WLEIS)_1. J Soc Sci Humanit.

[31] Nkongho N. The caring ability inventory. En: Strickland OL, Waltz CF. Measurement of nursing outcomes: Self care and coping. New York: Springer; 1990

[32] Cunha M, Duarte J, Cardoso A, Ramos A, Almeida V (2018). Caregiver skills inventory: factorial structure in a sample of portuguese participants. Millenium J Educ Technol Health.

[33] Douglas K (2011). When caring stops, staffing doesn’t matter: part II. Nurs Econ.

[34] Ma YL (2012). The research of human caring capacity and influence factors of nursing undergraduates.

[35] Wong CS, Law KS, Wong PM (2004). Development and validation of a forced choice emotional intelligence measure for Chinese respondents in Hong Kong. Asia Pac J Manage.

[36] Rodrigues N, Rebelo T, Coelho JV (2011). Adaptation of the Wong and Law Emotional Intelligence Scale (WLEIS) and analysis of its factorial structure and reliability in a Portuguese sample. Psychologica.

[37] Fukuda E, Saklofske DH, Tamaoka K, Lim H (2012). Factor structure of the Korean version of Wong and law’s emotional intelligence scale. Assessment.

[38] Acosta-Prado JC, Torres R, Torres G (2015). Characterization of emotional intelligence in Colombian managers. Univ Psychol.

[39] Chen SR, Zhang HM, Sun YS, Liang DD (2018). The investigation on influential factors of humanistic caring ability in nurses receiving standard training. Nurs Integr Tradit Chinese Western Med.

[40] Labrague L, Petitte D, Papathanasiou, I. Nursing students’ perceptions of their own caring behaviors: A multicountry study. International Journal of Nursing Knowledge. Advance online publication;20(4). 10.1111/2047-3095.12108.2015 10.1111/2047-3095.12108 26364825

[41] Cheng L, Liu Y, Ke Y (2016). Comparison of caring ability between Chinese and American nursing students. West J Nurs Res.

[42] Fjortoft N, Garrick D (2003). An assessment of pharmacists caring ability. J Am Pharm Assoc Japha.

[43] Wessel J, Larin H, Benson G, Brown B, Ploeg J, Williams R (2008). Emotional-social intelligence in health science students and its relation to leadership, caring and moral judgment. Internet J Allied Health Sci Pract.

[44] He J, Hu DY, Liu YL, Wu LF, Liu L (2016). Study of the effect of humanistic nursing care model wards in children caring ward school on the nurses’ caring ability. Chin Nurs Res.

[45] Bevis EO, Watson J (1989). Toward a caring curriculum: a new pedagogy for nursing. NLN Publ.

[46] Tomura M, Nagai M, Inaoka F (2005). The BSN curriculum based on human caring in the Japanese red cross Hiroshima college of nursing. Inter J Human Caring.

[47] Li X, Gao C, Liu H (2015). A comparative study of humanistic education curriculum in medical colleges in and abroad. China Med Pharm.

[48] Peng L, Ran S (2009). Present situation of medical humanities quality education in China. Northwest Med Educ.

[49] Mei L (2021). Research on humanistic care ability and humanistic curriculum of medical students in a comprehensive university in Xinjiang. Shihezi Univ.

[50] Arveklev SH, Wigert H, Berg L, Burton B, Lepp M (2015). The use a application of drama in nursing education–an integrative review of the literature. Nurse Educ Today.

[51] Yu HX (2019). Study on the correlation between emotional intelligence, coping style and job burnout of junior nurses.

[52] Wang YY, Liu H, Yuan LP, Wang GQ, Ye N, Jiang HJ (2019). Study on the correlation between nurses’ humanistic care ability and emotional labor and emotional intelligence. J Qiqihar Med Univ.

[53] Davies M, Stankov L, Roberts RD (1998). Emotional intelligence: in search of an elusive construct. J Pers Soc Psychol.

[54] Foji S, Vejdani M, Salehiniya H, Khosrorad RH (2020). The effect of emotional intelligence training on general health promotion among nurse. J Educ Health Promot.

[55] Inayat W, Khan MJ (2021). A study of job satisfaction and its effect on the performance of employees working in private sector organizations Peshawar. Educ Res Int.

[56] Nye CD, Su R, Rounds J, Drasgow F (2012). Vocational interests and performance: a quantitative summary of over 60 years of research. Perspect Psychol Sci.

[57] Luo SN, Hu YH, Wang HP, Pan XY (2020). Effect of emotional intelligence training on Job Burnout of clinical nurses. Nurs Rehabil.

[58] Esmaeili R, Alizadeh NR, Godarzian AH, Yousefi M (2015). The effect of emotional intelligence training on the quality of working life in nurses. J Health Sci.

[59] Burtson PL, Stichler JF (2010). Nursing work environment and nurse caring: relationship among motivational factors. J Adv Nurs.

[60] Wan F (2013). The change of humanistic caring ability in associate nursing students before and after clinical practice. Chin J Nurs Educ.

[61] Tai MG, Yang X, Guo L, Gao F, Wang K, Wu ZZ (2017). A study on the relationship between conflict management model and emotional intelligence of pediatric nurses in tertiary grade a hospitals. Chinese Nurs Manage.

